# Quantitative Analysis of Phase Response Enhancement in Distributed Acoustic Sensing Systems Using Helical Fiber Winding Technology

**DOI:** 10.3390/s25237289

**Published:** 2025-11-29

**Authors:** Yuxing Duan, Shangming Du, Tianwei Chen, Can Guo, Song Wu, Lei Liang

**Affiliations:** 1 Sanya Science and Education Innovation Park, Wuhan University of Technology, Sanya 572000, China; 307489@whut.edu.cn (Y.D.); shangming21@whut.edu.cn (S.D.); chentianwei@whut.edu.cn (T.C.); guocan@whut.edu.cn (C.G.); 307396@whut.edu.cn (S.W.); 2 National Engineering Research Center of Fiber Optic Sensing Technology and Networks, Wuhan University of Technology, Wuhan 430070, China; 3 School of Mechanical and Electrical Engineering, Wuhan University of Technology, Wuhan 430070, China; 4 School of Safety Science and Emergency Management, Wuhan University of Technology, Wuhan 430070, China; 5 School of Computer Science and Artificial Intelligence, Wuhan University of Technology, Wuhan 430070, China

**Keywords:** distributed acoustic sensing, helical fiber winding flexible hinge, seismic wave detection, phase response enhancement

## Abstract

In this paper, we investigate the physical mechanics of vibration wave detection in distributed acoustic sensing (DAS) systems with the aim of enhancing the interpretation of the quantitative wavefield. We investigate the nonlinear relationship of DAS gauge length and pulse width on the seismic wave response, and the result is explained by the trigonometric relationship of backscattered Rayleigh wave phases. We further demonstrate the influence of spiral winding on DAS performance and also build phase response models for P-waves and S-waves in helically wound cables. These models suggest that the winding angle controls the measurement interval spacing and the angle of wave incidence. Additionally, integration of structural reinforcement improves the amplitude response characteristics and SNR. The experimentally inspired results show, using simulations and field tests, that the same vibration sources can give helically wound cables with larger winding angles the largest phase amplitudes, which would substantially exceed that of straight cables. SNR increased significantly (approximately 10% to 30%). The efficacy of the method was also checked using experiments for different vibration amplitudes and frequencies. Such results provide evidence for the design and installation of fiber-optic cables for use in practical engineering applications involving safety monitoring.

## 1. Introduction

Distributed fiber-optic acoustic sensing (DAS) is an emerging technology that utilizes optical fiber as an ultralong linear array comprising tens of thousands of acoustic sensors to measure acoustic waves, vibrations, and continuous changes in real-time. Benefiting from its long sensing range, high spatial resolution, and strong immunity to electromagnetic interference, DAS shows great potential in oil and gas exploration [1], pipeline safety monitoring [2], geological hazard early warning [3], and infrastructure security [4].

The performance of a DAS system is not constant; the final monitoring effect depends on the precise settings of core system parameters. Key parameters include gauge length and pulse width, which determine spatial resolution, strain sensitivity, and signal-to-noise ratio. The pulse width defines the minimum resolvable unit along the fiber-optic cable, while gauge length determines the size of the virtual “acoustic sensor” for strain demodulation. There is a trade-off between the two: A shorter pulse width improves spatial resolution but reduces pulse energy, lowering the signal-to-noise ratio. Increasing the gauge length improves strain response sensitivity but tends to smooth out local high-frequency details, leading to signal distortion.

The gauge length is a crucial parameter in distributed acoustic sensing (DAS) surveys, directly influencing the signal-to-noise ratio (SNR) and resolution [5]. Dean [6] used a theoretical derivation to analyze the trade-off between the DAS gauge length and vibration wave SNR, as well as vertical seismic profiles, and thus formulated the most suitable gauge length relative to the wave speed and dominant frequency. He also demonstrated that using multiple gauge lengths can significantly improve data quality, although the effectiveness of this under nonlinear effects and real-world vibration conditions still requires verification.

Alfataierge [7] further quantified the relationship between DAS spacing and seismic profile fidelity through well-designed comparison experiments. However, the applicability of this criterion in medium- to high-frequency and complex media still requires verification. Luckie [8] showed that the spacing must be much less than half the vibration wavelength to balance resolution and directional accuracy; however, the signal-to-noise loss from ultrashort spacing and performance in the field remain to be confirmed.

Muir [9] studied the amplitude behavior of array fiber with respect to the DAS spacing, finding that without correcting the uncorrected spacing, amplitude errors could be up to 2-fold larger, and postulated wavefield correction and a hybrid array system to solve this issue. However, it remains a technical question to identify a suitable configuration of the sensing fiber array in terms of this system and the wavefield. Capdeville [10] studied the sensitivity of fixed-spacing DAS strain to microscale measurements and suggested adaptive spacing adjustments as needed. He also added that the effective fiber measurement with amplitude frequency implies a nonlinear relationship and that the global optimal range of spacing length is still to be determined.

Current research has not linked the core system parameters of DAS with vibration wave characteristics, leaving the coupling between fiber gauge length and wave feature response unexplained. The main challenge is to ensure accurate data acquisition while maximizing DAS efficiency. As DAS applications move from qualitative identification to quantitative analysis, the fragility of bare optical fibers has led to their routine encapsulation in optical cables for engineering use. However, the cable—acting as the core sensor—primarily detects axial strain, making it difficult to achieve comprehensive detection of complex wavefields. Selecting or designing the optimal optical cable for a given scenario is, therefore, a current research hotspot.

When buried optical fibers are used for seismic wave sensing, the fiber winding angle has a significant impact on the sensitivity of the DAS system. Spiral-wound cables, although having slightly lower maximum sensitivity than straight cables, provide a more balanced response to seismic waves from all directions [11]. Increasing the total length of spiral-wound fibers within the cable, as well as the winding density, can enhance sensitivity to environmental vibrations [12]. Al Hasani [13] has already reported that the fiber spiral winding angle can maximize the signal-to-noise ratio of such reflected waves, an improvement that remains to be found with tailored designs and manufacturability, as well as mechanical reliability.

In terms of surface deployment methods, Harmon [14] compared uncoupled, tension-coupled, and weighted soil-coupled schemes and showed that reliable phase information can be obtained without disturbing the ground surface. However, coupling strength and fixed-spacing coupling can still cause amplitude distortion and bandwidth limitations, so the spacing length and fiber deployment method require further optimization. Kuvshinov [15] theoretically analyzed the interaction between spiral-wound cables and plane seismic waves, suggesting that a smaller winding angle improves sensitivity to transverse waves. An optimal winding angle of about 30° for plastic cables can reduce the influence of Rayleigh waves. Eaid [16] emphasized that the ratio of spacing length to fiber pitch is critical for high-precision shear-strain imaging with full-waveform inversion, but the cable’s engineering and field verification still require improvement. Hudson [17] used wavefield coherence to assess the coupling between fiber and medium, demonstrating reliable extraction of amplitude data at millimeter-scale spacing lengths. This should broaden the use of amplitude-sensitive seismology, although the nonlinear relationship between spacing length and amplitude frequency remains unresolved.

While the aforementioned research established a foundation for applying fiber-optic cables in DAS systems, further refinements are necessary in theoretical models of spirally wound cables and the wavefield to achieve a high signal-to-noise ratio seismic wave perception. An in-depth investigation into the sensing performance and signal-to-noise ratio optimization of fiber winding angles and proportions is required.

Recent advancements in DAS have increasingly adopted helical fiber winding to counteract the axial bias present in straight cables, thereby improving omnidirectional sensitivity to seismic waves [18].

Indeed, previous studies [19] on shaped DAS fibers also described responses to microseismic moment tensors, which are used to describe interactions with elastic waves in subsurface locations. Other studies [20] have investigated directional sensitivities for surface seismic acquisition and proposed optimal winding angles to minimize the dominance of Rayleigh waves. Multi-objective optimization frameworks have also been proposed to adapt DAS parameters, such as helical designs, in order to improve signal fidelity in seismology [21].

However, these studies have mainly concentrated on qualitative improvements or simulations, often overlooking the comprehensive integration of nonlinear system parameters—such as gauge length and pulse width—that can lead to spatial filtering artifacts and amplitude distortions. Our proposed helical fiber wrapping sensitivity-enhancement model advances this by the following:

1. Quantitative phase response functions are also established to provide a more explicit link between wrapping geometry, wave incidence angles, and velocity components to predict amplitude behavior;
2. Describing nonlinearity in order to improve the choice of parameters in DAS for the optimization of the decisions for media–velocity inversion;
3. Offering empirical validation through active-source experiments that measure trade-offs, such as a 15–30% decrease in signal-to-noise ratio (SNR) for a 1–3-fold increase in amplitude gain.

Theoretically, this approach builds on previous models by combining both photoelastic and elastodynamic effects within a unified framework, providing engineering guidance for deploying sensing cables in applications such as geological hazard monitoring, where improved phase sensing can increase resolution without adding computational overhead to inversion workflows.

**Figure 1:**
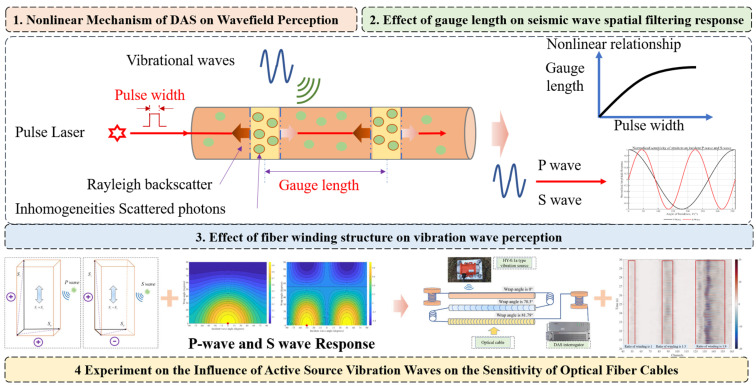
Summary of research contents.

This study investigates how spiral-wound optical fibers improve the phase response of DAS systems. The work, shown in Figure 1, advances step-by-step—from nonlinear mechanisms to structural effects and ultimately to experimental validation—through four interconnected components that build on each other to enhance quantitative wavefield sensing in fiber-optic technologies.

The first component, “Nonlinear Mechanism of DAS on Wavefield Perception,” explores the fundamental interactions between DAS system parameters—such as gauge length and pulse width—and seismic wave propagation. It explains how vibrational waves induce strain in the fiber via the Rayleigh backscattering of inhomogeneous photons from a pulsed laser. The section introduces a theoretical basis by demonstrating that these parameters induce nonlinear phenomena, such as spatial filtering and signal distortion, which should be addressed to correctly sense P-waves, S-waves, and surface waves in geophysical media.

The second component, “Effect of Gauge Length on Seismic Wave Spatial Filtering Response,” examines the spatial filtering characteristics imposed by gauge length. Illustrated by a nonlinear graph of gauge length versus pulse width, this section shows that longer gauge lengths increase strain sensitivity but smooth out high-frequency details, potentially causing amplitude deviations. It offers guidance on optimizing these parameters to balance signal-to-noise ratio (SNR) and resolution, which is essential for applications such as shallow subsurface exploration.

The third component, “Effect of Fiber Winding Structure on Vibration Wave Perception,” examines how helical winding enhances structural performance. The diagrams illustrate how winding angles change gauge length spacing and incident wave angles, which in the form of winding angles increases omnidirectional sensitivity and signal-to-noise ratio (SNR) in helically wound versus straight fiber. Instead of the pre-defined nonlinearity analysis outlined previously, this work employs geometric factors that increase phase responses in complex wavefields.

The fourth component, “Experiment on the Influence of Active Source Vibration Waves on the Sensitivity of Optical Fiber Cables,” verifies the theoretical models through outdoor testing. It outlines experimental configurations with active vibration sources that achieve the improvement in sensitivity in helically wound cables by confirming the increased amplitude characteristics, which is important for engineering applications like structural health monitoring and seismic hazard assessment.

Collectively, these components investigate the coupling between sensor spacing and pulse width, as well as the theory of fluctuation sensing in spiral-wound fibers. The objective is to provide theoretical support for optimizing DAS parameters and to establish a foundation for quantitative analysis and field testing of spiral-wound fibers, ultimately advancing DAS applications in geophysics, structural health monitoring, and related fields.

## 2. Nonlinear Mechanism of DAS on Wavefield Perception

The gauge length and pulse width are important interdependent parameters in a distributed acoustic sensing (DAS) system. Its ideal configuration must be designed to closely resemble the spectral characteristics of the measured vibration wave and meet application-specific demands related to spatial resolution and the signal-to-noise ratio (SNR), which can be a trade-off for the system. To address the necessary balance and optimization, a basic analysis of the fundamental wave propagation theory and the system response characteristics is first conducted.

### 2.1. Theory and Characteristics of Seismic Wave Propagation

Seismic wave propagation through strata underlies elastic-medium physical imaging, depending primarily on the medium’s elastic properties and density, and governed by elastic-wave theory. The wave equation, the core of this theory, accurately describes how vibrational energy varies in time and space. In a homogeneous isotropic medium, the wave equation for sound field propagation is given by Equation (1).

(1) $$\nabla^{2}u(x,t)-\frac{1}{v^{2}}\frac{\partial^{2}u(x,t)}{\partial t^{2}}=-s(x,t)$$

So, in this equation, u(x,t) is the wavefield disturbance at x and time t, and ∇^2^ is the Laplacian operator, which is the second-order spatial change in the wavefield. v is the wave propagation speed in the medium. The term s(x,t) is the source term in the wave equation, while the “initial energy” is usually part of the initial conditions. The term s(x,t) signifies the source term in the wave equation, whereas the initial energy is typically included in the initial conditions.

Although the Earth’s environment is complex and not uniform throughout its anisotropic regions, the equations that describe the joint propagation of compressional and shear waves must be a more sophisticated set of elastic wave equations. Thus, it is found that a more convenient mathematical framework exists by which to predict and simulate real wave phenomena.

Seismic wave types are essentially body waves and surface waves, depending on how they propagate and what their characteristics are. The speed of wave propagation plays a crucial role in how optical fiber perceives seismic wave packets. Physically speaking, body waves travel through Earth’s interior and comprise P waves and S waves. P-waves are longitudinal waves that show that the particles move in the direction the wave moves exactly the same way sound travels in air. They move the fastest and are the ones that sensors first track. Their velocity (V_p_) is obtained from the Lamé parameters λ and μ and the density ρ of the medium.

(2) $$V_{P}=\sqrt{\frac{\lambda +2\mu }{\rho }}$$

S-waves are transverse waves, with particle motion perpendicular to the direction of propagation. They move slower than P-waves and fail to propagate in an ideal fluid. The shear modulus μ and density ρ of the medium determine their velocity (V_s_).

(3) $$V_{S}=\sqrt{\frac{\mu }{\rho }}$$

Surface waves arise from the reflection and superposition of P-waves and S-waves at the Earth’s surface or at subsurface elastic boundaries. They primarily propagate along the Earth’s free surface or underground elastic interfaces, exhibiting slower energy attenuation and larger amplitudes. Thus, surface waves are a key focus in shallow subsurface vibration measurements.

Near the surface, wave velocities are relatively low, allowing refracted vibrations to transfer energy into low-velocity layers and accumulate as “trapped waves.” Constructive interference occurs between the waves to generate surface waves. More precisely, interference resulting from P-waves and S-waves brings Rayleigh waves—colloquially known as ground roll. Rayleigh waves generate elliptical motion of particles in the vertical plane similar to ocean waves, and VR depends on both P-wave and S-wave velocities [22].

This is shown in Formula (4).

(4) $$V_{R}=V_{S}\cdot \frac{0.862+1.14\nu }{1+\nu }$$

As mentioned in Formulas (5) and (6), the Poisson’s ratio, ν, is derived from Formulas (2) and (3).

(5) $$\nu =\frac{\lambda }{2(\lambda +\mu )}$$

(6) $$\frac{V_{P}^{2}}{V_{S}^{2}}-2=\frac{\lambda }{\mu }$$

To complete the relationship between Rayleigh wave velocity and the velocities of longitudinal and transverse waves, a velocity ratio relationship is established in conjunction with Poisson’s ratio, as shown in Formula (7).

(7) $$\nu =\frac{{\left(V_{P}/V_{S}\right)}^{2}-2}{2({\left(V_{P}/V_{S}\right)}^{2}-1)}$$

Based on a representative isotropic medium in a homogeneous state, the ratio of P-wave strain energy (W_p_) to S-wave strain energy (W_s_) in Rayleigh waves can be calculated by integrating across a semi-infinite expanse. According to the theory of Aki & Richards [23], this ratio can be expressed as

(8) $$\frac{W_{P}}{W_{S}}=\frac{4\alpha \beta -k^{2}-\beta^{2}}{k^{2}+\beta^{2}}$$

where k is the Rayleigh wave number, V_p_ and V_s_ are the P-wave and S-wave velocities of the medium, respectively, and V_R_ is the Rayleigh wave velocity. The coefficients α and β represent the exponential depth decay of the P-wave and S-wave components, determined by the wave number and corresponding body wave velocities. Specifically, $\alpha =k\sqrt{1-(V_{R}/V_{P}{)}^{2}}$ and $\beta =k\sqrt{1-(V_{R}/V_{S}{)}^{2}}$.

These analyses reveal that the internal structure of Rayleigh waves is determined by the P-wave velocity, S-wave velocity, and Poisson’s ratio, which collectively govern the distribution of wave energy. Employing distributed acoustic sensing to sample surface waves generated by active or passive sources at a high density and then analyzing their amplitude characteristics to invert the physical state of the surveyed structure is a current research focus. Consequently, determining how to utilize fiber-optic technology to represent and capture these features accurately is a key direction for the subsequent work in this paper.

Research manuscripts reporting large datasets that are deposited in a publicly available database should specify where the data have been deposited and provide the relevant accession numbers. If the accession numbers have not been obtained at the time of submission, please indicate that they will be provided during the review process. They must be provided before publication.

Interventional studies involving animals or humans, as well as other studies that require ethical approval, must list the authority that provided approval and the corresponding ethical approval code.

### 2.2. Nonlinear Relationship Between Gauge Length and Optical Pulse

The gauge length and pulse width, key parameters of a DAS system, greatly influence how nonlinear effects are perceived and measured. Studying how these two factors interact nonlinearly enhances understanding of the system’s response under imperfect coupling conditions, providing a theoretical basis for improving fiber-based vibration measurements.

In distributed acoustic sensing (DAS) systems, the link between pulse width and measurement range mainly affects spatial resolution and signal strength. Spatial resolution is given by the formula Δz = c × τ/2n, where τ is the pulse width, c is the speed of light, and n is the refractive index. The range of measurements depends upon signal strength decreasing with distance and requires longer pulses for compensation. The desired area expansion as the signal range increases, the pulse width of the ideal (x) goes up, but smaller pulses give better spatial resolution. Typically, the optimal pulse width is the shortest possible that still ensures adequate signal strength for a measurement length L. Narrower pulses improve resolution. In contrast, wider pulses increase signal strength, making them ideal for longer distances.

In the experiment, to figure out the spatial resolution of Δz, the optical pulse width τ was set to 50 ns. The gauge length L_g_ is usually an integer multiple of Δz or is determined by the system algorithm, and was set to 5 m by the DAS design factor k = 4. The AP-sensing N52-R50 interrogator was implemented at a 10,000 Hz sampling frequency with a 1.25 m spatial sampling interval.

(9) $$L_{g}=k\cdot \Delta z$$

**Figure 2:**
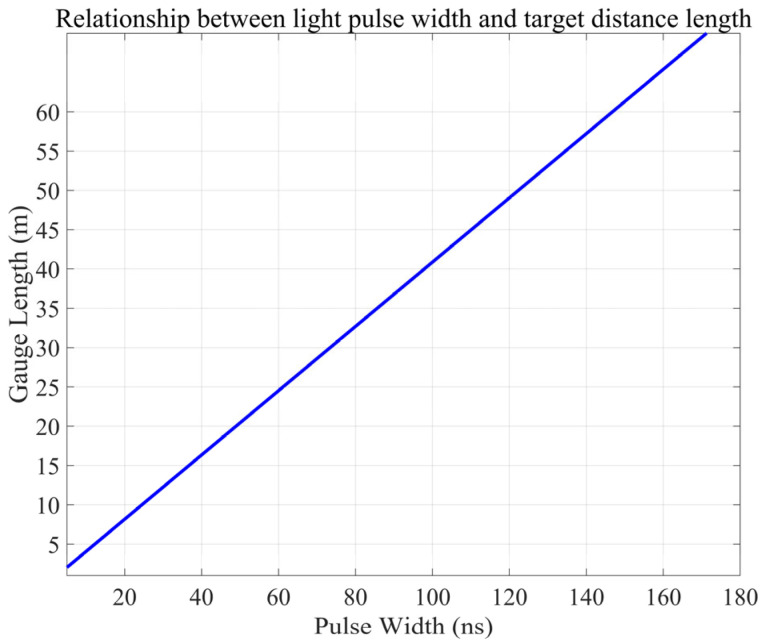
Pulse width and mark spacing length function relationship.

Figure 2 demonstrates the approximately linear comparison of the optical pulse width τ and gauge length L in a DAS system. As τ varies from 0 to 200 ns, L runs from 0 to ~80 m using an estimated L ≈ 0.40 τ. When τ is too short, far-end Rayleigh backscattering cannot temporally overlap with near-end scattering, and interference signal intensity is greatly reduced rapidly, with measurement accuracy declining rapidly with increasing range. Alternatively, excessively long pulses add more background noise and can lead to overlapping multiple scattering, both of which contribute to lowering signal-to-noise ratio while also increasing system complexity and error sources, sometimes making signal interpretation difficult. Moreover, in complex geological or high-strain environments, incomplete fiber–medium coupling and variations in the scattering coefficient can produce local deviations from this linear trend.

To balance dynamic range and measurement accuracy, the pulse duration τ should be chosen based on the target range. For short distances, use shorter pulses to boost spatial resolution. For longer distances, increase τ and implement noise-reduction and multiple-scattering-correction algorithms to ensure dependable wavefield sensing and medium-parameter inversion across various complex conditions.

## 3. The Effect of Gauge Length on Seismic Wave Spatial Filtering Response

### 3.1. Response of a Straight Optical Fiber to Multi-Angle Vibration Waves

Primary (P) waves are compressional elastic waves whose propagation can be precisely described by elastodynamic theory. In this framework, the wavefield is defined by a scalar potential, digamma, and a vector potential, al ϕ. A purely P-wavefield is inherently irrotational, meaning that the vector potentials $\xi_{p}$ and $\phi_{P}$ responsible for shear or rotational motion vanish. Consequently, the derivation of particle motion and medium deformation in Equation (10) can be carried out using only the scalar potential.

(10) $$\phi_{P}=A_{0}\frac{\alpha }{\omega^{2}}e^{i(k\cdot x-\omega t)}$$

A particle vibration velocity vector is a fundamental kinematic parameter that describes how a medium responds to a P-wave effect, describing the instantaneous path of each target area as the wave propagates. In mathematical notation, this vector form is Equation (11), with amplitude factor A_0_, representing the initial wave strength, a complex exponential number that indicates how an action plane wave propagates in space and time, and a vector in a unit direction. The direction vector resolves the particle vibration along the wave propagation direction into Cartesian coordinates based on the incidence angle δ: the cos δ term represents the vertical (*z*-axis) component, and the sin δ term represents the horizontal plane component. Here, Ω denotes the azimuthal angle in the horizontal (x–y) plane, specifying the wave’s horizontal projection orientation relative to the coordinate axes. This follows the standard convention in spherical coordinate decompositions for plane waves, where δ is the incidence angle from the *z*-axis and Ω defines the rotational position around it.

(11) $$v_{p}=A_{0}[\sin\delta \cos\Omega \hat{x}+\sin\delta \sin\Omega \hat{j}+\cos\delta \hat{z}]e^{i(k\cdot x-\omega t)}$$

The *Z*-axis of the particle vibration velocity is given by Equation (12) and is proportional to cos δ, which is the cosine of the incidence angle.

(12) $$v_{z}=A_{0}\cos\delta e^{i(k\cdot x-\omega t)}$$

The impact of a vibrational wave on an elastic medium produces particle displacement, deformation, and motion. According to the fundamentals of elasticity theory, the function ω(z, t) describes the displacement at a given point along the fiber’s axial direction. In Equation (13), the strain component $\varepsilon_{z}$ is defined as the spatial derivative of the axial displacement w with respect to the z direction in a Cartesian coordinate system.

(13) $$\varepsilon_{z}=\frac{\partial w}{\partial z}$$

The standard method for obtaining the temporal and spatial derivatives of harmonic plane waves passing through a medium is through transformations into the frequency and wavenumber domains.

(14) $$\varepsilon_{z}=\frac{ik_{z}}{-i\omega }v_{z}$$

In the transformed domain ∂/∂t is multiplication by –iω, and ∂/∂z of the axial displacement w is multiplication by ik_z_. In this mapping, strain, first given as displacement, corresponds to the particle vibration velocity, v_z_ (Equation (14)). A value for v_z_ and a value from the axial wavenumber k_z_ = ω/(c cos δ) (where c is the phase velocity and δ is the incidence angle) can be substituted to make the axial strain ε_z_ less complex by performing frequency and wavenumber transformation. The angular frequency ω is canceled, and the trigonometric factors are combined to form the terminal analytical expression for ε_z_ (Equation (15)).

(15) $$\begin{array}{l} \varepsilon_{z}^{DAS}=\frac{ik_{z}}{-i\omega }v_{z}=\frac{(\omega /\alpha )\cos\delta }{-\omega }(A_{0}\cos\delta e^{i(k\cdot x-\omega t)})\\ =\frac{\cos\delta }{-\alpha }(A_{0}\cos\delta e^{i(k\cdot x-\omega t)})=-\frac{A_{0}}{\alpha }\cos^{2}\delta e^{i(k\cdot x-\omega t)} \end{array}$$

**Figure 3:**
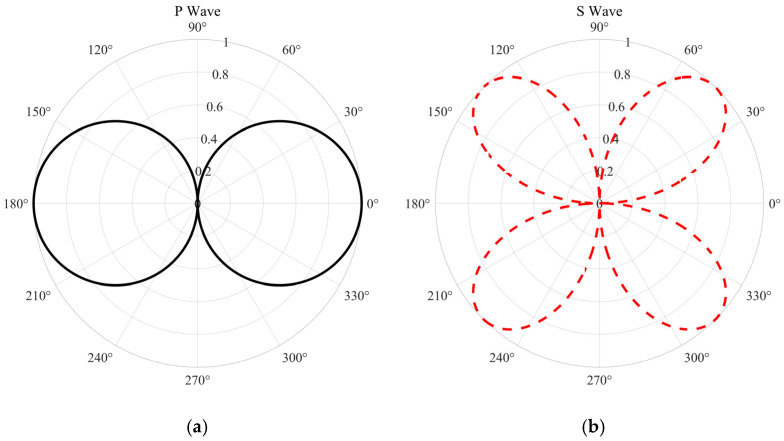
Optical fiber response curves to two types of seismic waves at varying incidence angles. (**a**) P-waves. (**b**) S-waves.

Equation (16) provides the axial strain rate measured by the DAS fiber, which is proportional to the square of the cosine of the incidence angle, cos^2^δ, according to its amplitude. Now, as shown by the derivation results shown in Figure 3a for a given incident P-wave, the dependence of particle vibration velocity and axial strain on incidence angle is fundamentally different.

(16) $$\varepsilon_{z}^{DAS}=-\frac{A_{0}}{\alpha }\cos^{2}\delta e^{i(k\cdot x-\omega t)}$$

An incident S-wave is a shear wave whose particle motion is perpendicular to its direction of propagation. To simplify the analysis, the incident S-wave is decomposed into a vertically polarized SV component and a horizontally polarized SH component.

The motion of the SH wave only occurs in the horizontal plane, with no vertical (*z*-axis) velocity or strain applied. The particle vibration velocity vector is the medium’s kinematic response to the SV wave. Its vector expression (Equation (17)) precisely represents the instantaneous motion at any point in the medium, comprising an amplitude term, a propagation term, and a direction vector. The direction vector is orthogonal to the shear-wave nature of the SV wave’s propagation direction and is resolved into Cartesian components based on the incidence angle δ.

(17) $$v_{s}=A_{0}[-\cos\delta \hat{x}+\sin\delta \hat{z}]e^{i(k\cdot x-\omega t)}$$

By substituting the SV wave’s axial velocity component and the axial wavenumber k_z_, and using the shear wave velocity β, then applying algebraic simplification and trigonometric identities, one obtains the analytical expression for axial strain (Equation (18)). As shown in Figure 3b, the wave’s directional response is governed by the sin(2δ) term.

(18) $$\varepsilon_{z}^{DAS}=\left(\frac{(\omega /\beta )\cos\delta }{-\omega }\right)(A_{0}\sin\delta e^{i(k\cdot x-\omega t)})=-\frac{A_{0}}{2\beta }\sin(2\delta )e^{i(k\cdot x-\omega t)}$$

**Figure 4:**
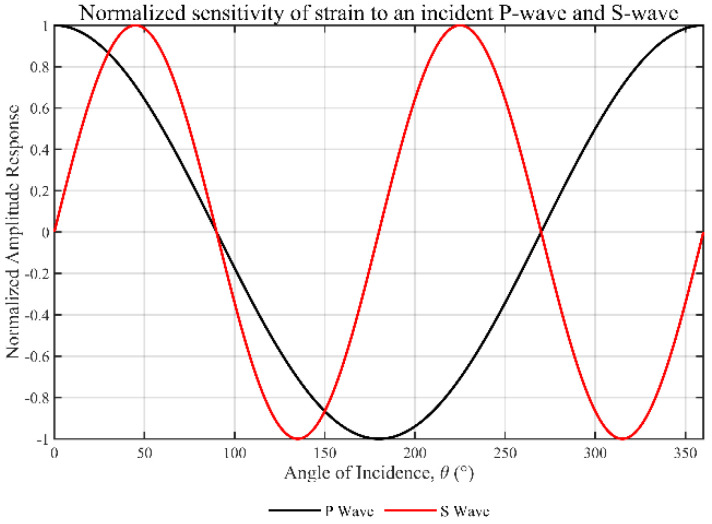
Fiber-optic response curves for P-waves and S-waves at different incident angles.

As shown in Figure 4, based on the preceding analysis, we establish a theoretical model for the normalized amplitude response of a straight-laid fiber sensor to the P-wave and S-wave as a function of incidence angle θ. In this model, θ equals zero degrees when the wave propagates parallel to the fiber axis. The P-wave, represented by the black curve, exhibits a dipole behavior as cosθ, with maxima at 0° and 180°, and zeros at 90° and 270°, indicating that it is not tolerant to normal incidence. The red response curve of the S-wave exhibits a quadrupole sin2θ pattern, with zeros at 0°, 90°, 180°, and 270°, and peaks at 45°, 135°, 225°, and 315°, resulting from the increase in axial deformation caused by the obliquely incident shear waves. This anisotropic behavior simultaneously showcases the blind edges characteristic of a fiber, while also providing a platform for distributed acoustic sensing to discriminate P-wave responses from S-wave responses, intercept the direction and polarization of seismic wave incidence, and to more effectively observe the complex wavefields on a multi-directional or helical cable.

### 3.2. Spatial Filtering Mechanism and Wavefield Response of DAS Systems

Distributed acoustic sensing measures phase changes caused by external disturbances along the fiber. This is achieved by integrating the local strain over each sensing segment and then calculating the difference between adjacent segments, as shown in Equation (19).

(19) $$\varepsilon_{zz}^{DAS}=\frac{1}{GL}\int_{z-\frac{GL}{2}}^{z+\frac{GL}{2}}\varepsilon_{z}^{DAS}dz^{\prime }$$

Extending previous investigations of fiber response to wavefields, and accounting for segment length with wavelength sensitivity, substituting Equation (16) into Equation (19) produces Equation (20).

(20) $$\varepsilon_{zz}^{DAS}=\frac{1}{GL}\int_{z-\frac{GL}{2}}^{z+\frac{GL}{2}}\left(-\frac{A_{0}}{\alpha }\cos^{2}\delta e^{i(k_{z}z^{\prime }-\omega t)}\right)dz^{\prime }=-\frac{A_{0}}{\alpha GL}\cos^{2}\delta e^{-i\omega t}{\left[\frac{1}{ik_{z}}e^{ik_{z}z^{\prime }}\right]}_{z-\frac{GL}{2}}^{z+\frac{GL}{2}}$$

In this case, GL is the DAS gauge length. where z′ denotes the vector, the position along the fiber axis is the integration variable. A_0_ is the amplitude of the wave, α is the phase velocity, δ is the angle of the wave propagating direction and the *z*-axis, and k_z_ is the axial component of the wave vector. Then, use the above from Euler’s equation, and all terms, as well as the addition of the sinc function, to obtain Equation (21). DAS gauge length and wave parameters are included in Equation (21). That indicates the DAS wavefield response function.

(21) $$\varepsilon_{zz}^{DAS}=-\frac{A_{0}}{\alpha GL}\cos^{2}\delta e^{-i\omega t}\frac{1}{ik_{z}}e^{ik_{z}z}\left(2i\sin\left(\frac{k_{z}GL}{2}\right)\right)=-\frac{A_{0}}{\alpha }\cos^{2}\delta e^{i(k_{z}z-\omega t)}\sin c\left(\frac{k_{z}GL}{2}\right)$$

This study reveals that DAS measurements do not directly represent true strain. The $\left(-A_{0}/\alpha \right){cos}^{2}\delta e^{i(k_{z}z-\omega t)}$ term exactly describes the true strain induced by an incident plane wave along the fiber axis. The $\sin c\left(k_{z}GL/2\right)$ term captures the inherent spatial averaging effect of the DAS gauge length GL; namely, the spatial average of the instantaneous $\varepsilon_{inst}=\varepsilon_{avg}\sin c\left(x\right)$ over the gauge length. Mathematically, this averaging is equivalent to a spatial filtering effect of a sinc function.

**Figure 5:**
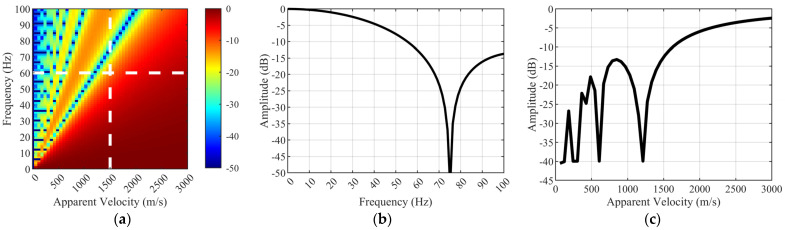
DAS fiber-optic spectral response over a 20 m spatial range. (**a**) Spectral response of 20 m gauge. (**b**) Cut at v = 1500 m/s. (**c**) Cut at f = 60 Hz.

Figure 5 shows the sinc spectral response for a 20 m gauge length, with velocity on the horizontal axis and frequency on the vertical axis. Color variations indicate the filtering effect of the sinc function on DAS strain rate data, expressed in decibels.

Two white strokes are visible in the figure to illustrate this phenomenon. A dashed line is the cut through the response surface at v = 1500 m/s (Figure 5b). The frequency spectrum exhibits a significant notch at ~75 Hz within the frequency range, where the amplitude drops sharply on both sides, resulting in a zero crossing. Note that all measured strain signals of the wave propagating at 1500 m/s will exhibit considerable attenuation above 60 Hz. The constant frequency cut at 60 Hz, also known as the white dot frequency (see also Figure 5a), is visible in Figure 5c. This indicates that strain signals from any wave traveling at apparent velocities below ~1500 m/s will be significantly attenuated in our measurements.

## 4. Impact of Fiber Winding Structure on Vibration Perception

Past studies have investigated the response behavior of DAS fiber to longitudinal and transverse waves, examined the trigonometric influence of wave incident angles on straight fiber coupling efficiency, and analyzed the spatial filtering effects of DAS pulse width and gauge length on wave measurements. In practical engineering applications, optical fiber cables serve as direct physical contact sensors, making it crucial to enhance sensing efficiency and sensitivity.

The principal mechanism of helically wound fiber fundamentally changes the effective gauge length for response. This section primarily focuses on the influence of helically wound fibers on DAS, introducing a sensing model for the helical fiber’s response to vibration waves. Starting with the effects on DAS gauge length from fiber winding angle, this analysis will examine the relationship between fiber winding angle and incident waves, and verify theoretical findings from the simulation. These theoretical results provide a foundation for designing sensing cables.

### 4.1. The Effect of Fiber Winding Angle on the Increase in Marking Distance

**Figure 6:**
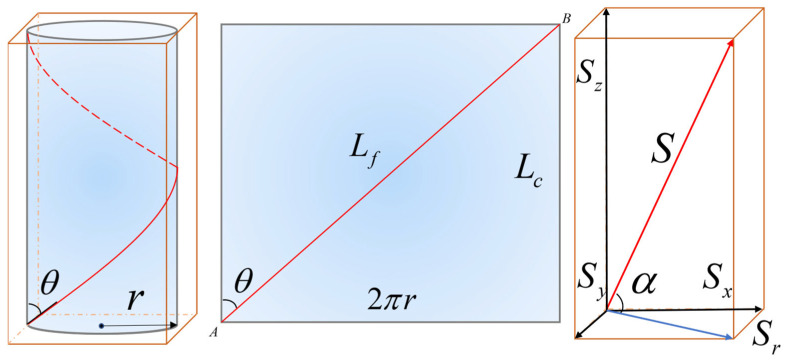
Fiber Bragg Grating Single-Period Axial Sensitivity Geometric Model.

To more accurately describe the geometry of helically wound optical fiber, a geometric model of the fiber winding on the cable core within one pitch is established, as illustrated in Figure 6. In this model, points A and B represent the start and end points of one complete winding pitch of the fiber on the cable core. The parameter r denotes the radius of the cable core. The length of the cable from point A to point B is the pitch length, while L_f_ represents the actual length of the fiber between points A and B.

The helical winding angle θ is defined as the angle between the fiber and the cable axis. θ = 0° denotes a straight fiber aligned with the axis (minimal winding), while θ approaching 90° indicates a tightly wound fiber, which increases the effective length and enhances interaction with the incident wave. Based on the geometric conditions of the model, the relationship between the cable pitch length $L_{c}$ and the actual fiber length $L_{f}$ can be expressed as follows:

(22) $$L_{c}=L_{f}\cos\theta$$

From the intrinsic properties of optical fiber, it is possible to conclude that the relation between axial strain and phase change for optical fiber is given by Formula (23).

(23) $$\Delta \phi =knL_{f}\varepsilon_{z}\left(1-\frac{n^{2}}{2}[P_{12}-\nu (P_{11}+P_{12})]\right)$$

In this equation, n represents the refractive index of the fiber, k = 2π/λ is the wave number, and λ is the average wavelength of the laser in a vacuum within the system. The parameter L denotes the length of the fiber under perturbation, while ε represents the axial strain of the fiber. The term v is Poisson’s ratio, and P_11_ and P_12_ are the photoelastic coefficients of the fiber. As the helical winding angle θ increases, the cosine of θ decreases, resulting in a longer effective length L_f_. The increase in winding angle θ creates a longer fiber path, which enhances interaction with acoustic waves and consequently improves signal strength.

### 4.2. The Relationship Between Wrapping Angle and Incident Wave Phase Shift

**Figure 7:**
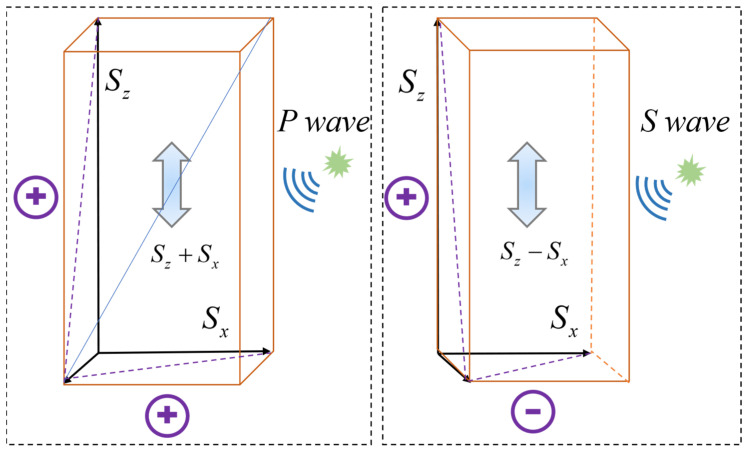
Diagram of fiber-optic cable changes under longitudinal and transverse wave action.

**Figure 8:**
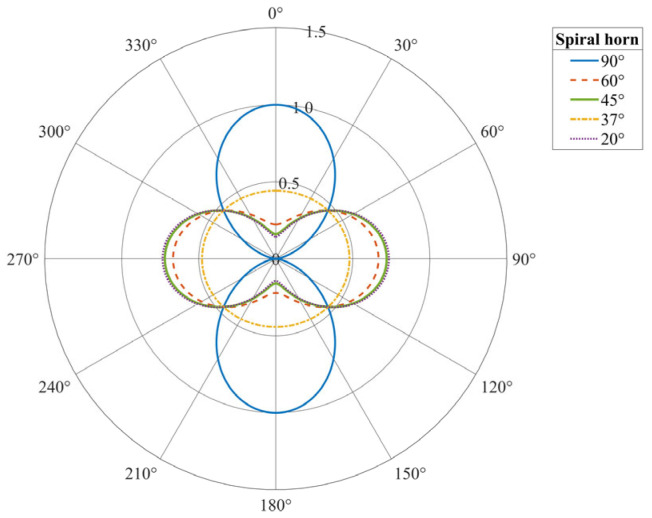
P-wave Response for Various Wrapping Angles.

The total response generated by vibration wave impacts on the optical fiber includes its interaction with equivalent segments decomposed along orthogonal axes. As shown in Figure 7 and Figure 8, the axial segment of a single fiber cycle can be equivalent to a linear distribution with uniform sensitivity along the axial direction. The sensitivity variation at any point on the helically wound fiber is described using parametric equations, as presented in Equations (24)–(26).

(24) $$S_{z}=S\cos^{2}\alpha$$

(25) $$S_{r}=S\sin^{2}\alpha$$

(26) $$S_{x,y}=\frac{1}{2}S\sin^{2}\alpha$$

where S represents the total sensitivity, and S_z_, S_r_, S_x_, and S_y_ represent the sensitivities along the *Z*-axis, in the plane perpendicular to the *Z*-axis, along the *X*-axis, and along the *Y*-axis, respectively.

Figure 7 demonstrates that longitudinal wave components generate significant and superimposable axial strain signals at both ends of the differential measurement, resulting in a strong response. In contrast, transverse wave components cannot produce axial strain in the fiber due to their vibration direction being perpendicular to the fiber axis. Consequently, their contributions at both ends are zero, leading to a final response of zero as they are effectively “canceled out.”

Next, we introduce the DAS fiber axial strain rate Formula (15) from Section 3.1, as presented earlier, and derive Equation (27), which establishes that the fiber sensitivity response intensity characteristics are proportional to the square of the cosine of the longitudinal wave incident angle, cos^2^δ.

(27) $$D_{P}(\alpha ,\delta )=\cos^{2}\alpha \cos^{2}\delta +\frac{1}{2}\sin^{2}\alpha \cos^{2}\delta$$

Similarly, the fiber response intensity characteristics are proportional to the sine of the transverse wave incident angle sin2δ, leading to the derivation of Equation (28).

(28) $$D_{s}(\alpha ,\delta )=\left|\sin2\delta \cos^{2}\alpha \right|-\left|\begin{array}{c} 1\\ 2 \end{array}\sin^{2}\alpha \sin2\delta \right|$$

The winding angle β of helically wound fiber and the angle δ between the vibration wave velocity and the fiber axis jointly determine the strain variations resulting from the interaction between vibration waves and helically wound fiber, thereby affecting the phase signal changes within the DAS fiber. The state β = 90°, corresponding to α = 0°, is defined as the straight fiber condition.

Combining the established relationship between fiber phase change and strain from Equation (23), along with the previously studied DAS fiber gauge length measurement parameters, we rederive ε = dϕ/n*_eff_* GLk, where dϕ represents the total change in the DAS fiber-optical phase in radians. GL is the fiber length used by the DAS system for phase differential measurement. The parameters $\lambda_{c}$ and $\lambda_{g}$ represent the first Lamé parameters of the optical cable and formation, respectively, while $N_{c}$ and $N_{g}$ represent the shear moduli of the optical cable and formation, respectively, which are also the second Lamé parameters.

The final relationship formula for the DAS fiber phase under P-wave vibration coupling is shown in Equation (29).

(29) $$\phi =n_{eff}GLk\left(\cos^{2}\delta \sin^{2}\beta +\sin^{2}\delta \cos^{2}\beta \frac{\lambda_{g}+2N_{g}}{2(\lambda_{c}+N_{c}+N_{g})}\right)$$

The interaction angles on vibration waves in a helically wound fiber are more complex because the spatial curvature of the fiber causes the angle between the vibrations and specific segments of the local fiber to vary and keep changing. Simulation results are shown in Figure 8, highlighting the helical fiber’s response to longitudinal waves at varying incident angles. Notably, the phase response difference is very significantly based on the winding angle, as illustrated by the varying colors.

**Figure 9:**
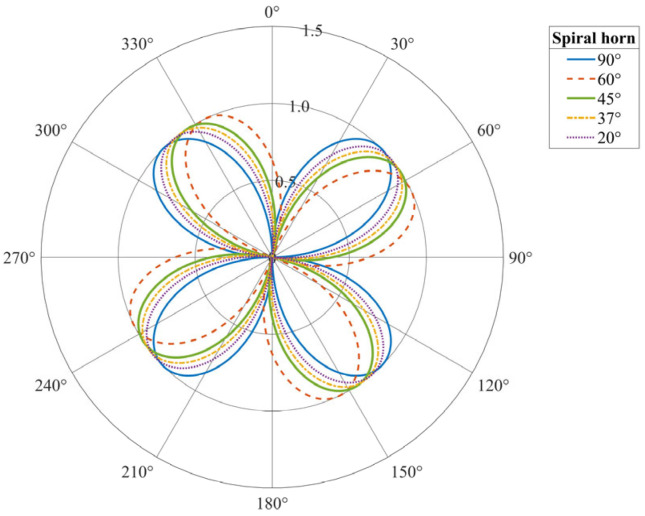
Diagram of a fiber-optic cable undergoing changes under the action of longitudinal and transverse waves.

As shown in Figure 9, the phase response variations in helical wound fiber are represented by different colors under transverse wave action at various incident angles.

The sensitivity response characteristics of the helically wound fiber-optic sensing cable to P-waves and S-waves were examined. The sensitivity varies as a function of both the incident wave angle and the fiber wrapping angle.

The response pattern of the sensing cable for P-waves is single-peak. Maximum sensitivity, observed with warm-colored regions, is reached when the incident wave front is perpendicular to the cable axis at normal incidence and is predominantly concentrated at smaller wrapping angles. As the incident angle deviates toward grazing directions of ±90 degrees or the wrapping angle increases, sensitivity monotonically decreases, indicating that this sensor achieves optimal detection performance for normally incident compressional waves.

**Figure 10:**
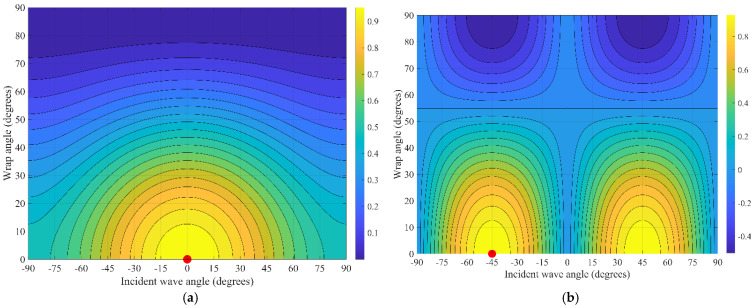
The sensitivity of a helical fiber-optic cable is related to the incidence angles of P(A) and S(B) waves and the fiber winding angle. (**a**) P-wave response contour plot (maximum). (**b**) S-wave response contour plot (maximum).

As shown in Figure 10, unlike the response of the cable to P-waves, the reaction to S-waves exhibits a dual-peak or quadrupole directional pattern. Maximum sensitivity is observed at ~±45°, whereas a blind zone is found in the direction where the P-wave response is most excellent at 0°. Such dual-peak sensitivity is most prominent at small wrapping angles. The S-wave response map also shows a shift in the response pattern or a reversal near a wrapping angle of approximately 55°.

### 4.3. Simulating Phase Change in Fiber Winding Under Vibration Waves

This section simulates the response of a distributed acoustic sensing (DAS) system to a specific vibration source, analyzing the dynamic phase response characteristics of different helically wound high-sensitivity optical cables under sinusoidal vibration excitation. The scenario includes a 50 m fiber with 10 m isolation loops at each end that do not contact the source but serve for signal buffering and noise simulation. The central 30-m segment forms the effective sensing region.

The simulation uses a custom MATLAB R2024b script to leverage its numerical computing environment for efficiency and ease of use. Wave propagation from the source is modeled with a one-dimensional time domain finite difference scheme, using a sinusoidal excitation with a frequency of 30 Hz, a speed of 500 m/s, an attenuation factor of Q = 3, and medium attenuation. The discretization employs a 0.01 m spatial grid (matching the Rayleigh scattering interval) and a 10 µs time step for numerical stability. Fiber strain response is obtained by numerically integrating the incident wavefield along the helical path length $L_{f}$ using Equation (22). Angle-dependent sensitivity is introduced via Equations (27) and (28). Phase change is then derived with Equation (23), where refractive index n = 1.5, wavelength λ = 1550 nm, and photoelastic coefficients P_11_ = 0.121, P_12_ = 0.270.

Based on this configuration, the DAS system employs a 1550 nm wavelength light source that emits 20 ns pulsed optical signals at a frequency of 1000 Hz. The optical fiber has a refractive index of 1.5, with Rayleigh scattering centers spaced at 0.01 m intervals and transmission loss of 0.25 dB/km. The system acquires data at a sampling rate of 100 MHz and processes the return signals from 100 consecutive optical pulses. During demodulation, the spatial sampling interval is set to 1.25 m.

**Figure 11:**
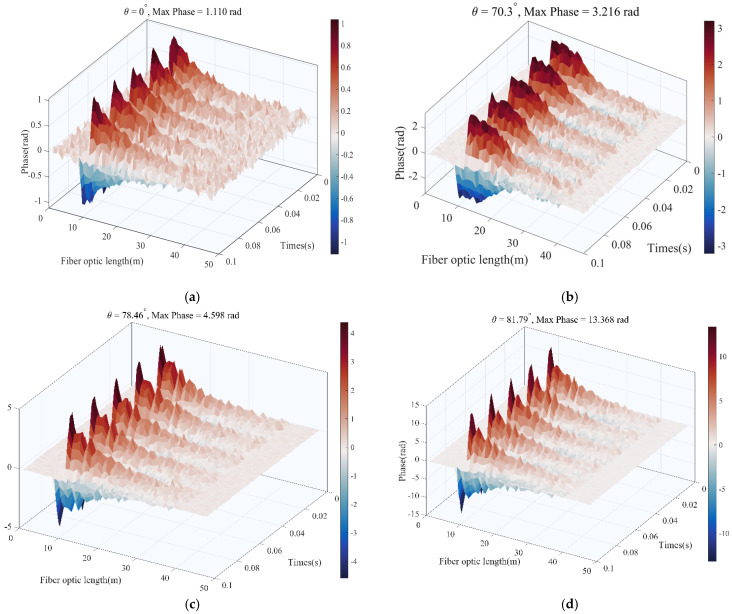
Sensitivity and angle change trend of spiraled optical fiber cable after wave vibration. (**a**) Fiber-optic cable helix winding ratio is 1. (**b**) Fiber-optic cable helix winding ratio is 3. (**c**) Fiber-optic cable helix winding ratio is 5. (**d**) The fiber-optic cable has a helix winding ratio of 7.

The fiber-optic cable helical winding angles are chosen as 0°, 70.3°, 78.46°, and 81.79°, respectively, with winding ratios of Lr = 1, Lr = 3, Lr = 5, and Lr = 7, as shown in Figure 11. As a parameter, the winding ratio Lr = 1/cos θ, which measures the actual length of fiber deployment in one unit of axial length, is an essential parameter for achieving enhancement in DAS phases.

**Figure 12:**
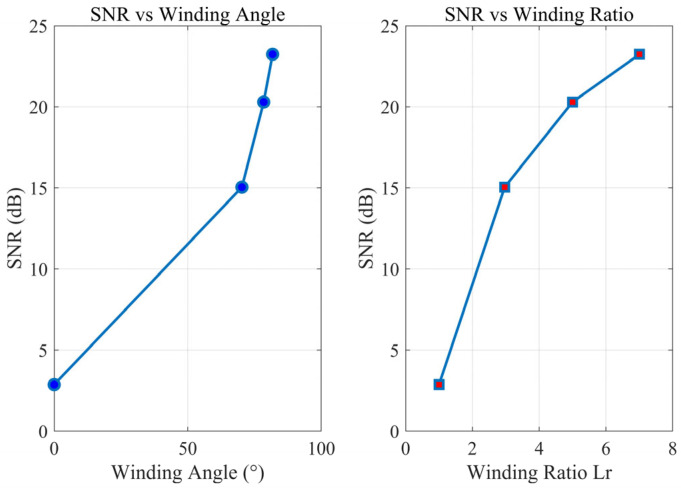
The relationship between signal-to-noise ratio, wrap angle, and proportion.

The simulation results for four different winding angles were used to calculate their signal-to-noise ratios (SNRs), as shown in Figure 12. Overall, the SNR increased markedly with larger Lr. The curve’s slope is steep at low Lr and flattens at high Lr, indicating that while a higher winding ratio continues to boost SNR (phase enhancement), the marginal gain diminishes.

Signal power and noise power were obtained statistically. Signal power was computed as the mean square value of the signal components across all fiber positions, and noise power was based on a −20 dBW white Gaussian noise (WGN) level. The SNR in decibels (dB) was then calculated as SNR = 10 log_10_(signal power/noise power), as shown in Table 1.

Based on the above simulation analysis results, the 20 dB improvement brought by the winding ratio Lr = 7 is because it amplifies the phase signal of the optical fiber when Lr = 1 by about 10 times. The strain amplification effect and length amplification effect produced by the optical fiber spiral winding collectively increase the signal phase, thereby improving the signal-to-noise ratio (SNR).

In this study, the propagation and attenuation characteristics of vibration waves in geological media are accurately replicated, along with the influence of vibration field modulation on the behavior of the fiber phase. For the first time, the research introduced a model to simulate the sensitivity enhancement and geometric effects introduced by a helical winding. These findings also offer significant lessons in comprehending and evaluating design principles for DAS systems with helically wound cables, providing a theoretical underpinning and data groundwork that can be integrated with other methods for field testing.

## 5. Experiment on the Influence of Active Source Vibration Waves on the Sensitivity of Optical Fiber Cables

### 5.1. Experimental Measurement and Deployment Plan

This study aims to verify the practical effectiveness of helical winding technology in terms of increasing signal amplitude and signal-to-noise ratio, considering the integration of theoretical models based on previous work in the field of engineering. The test site corresponds to a typical shallow burial engineering environment. An eccentric vibrator, HY-0.1a, is used as the active vibration source, with a power rating of 30 W. The frequency at which it vibrates can be varied from 0 to 60 Hz. The vibration source is mounted on a base using bolts, and the base is securely connected to the soil through four anchoring pins. The rotating eccentric mass generates sinusoidal excitation, with vibrations coupled to the soil through the base.

**Figure 13:**
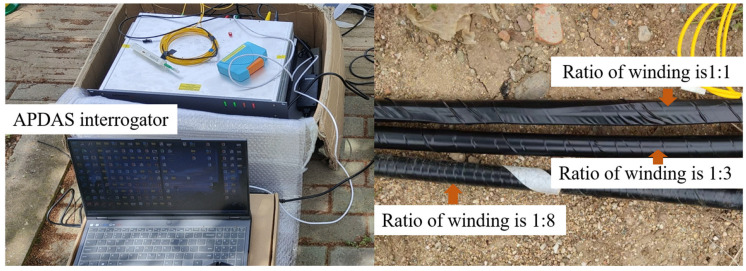
Fiber-optic sensor and DAS experimental test site diagram.

As shown in Figure 13, treating the cable as an integrated unit without considering the influence of internal structure on measurement results, the experiment utilizes helically wound cables simulated in the previous section. The core substrate consists of a 9 mm diameter polyurethane rod, which provides superior flexibility compared to conventional glass fiber materials. Bend-resistant optical fibers are helically wound around the polyurethane rod exterior with winding ratios of 1, 3, and 8, respectively. Black heat-shrink tubing is added to protect the reliability of optical signal transmission.

**Figure 14:**
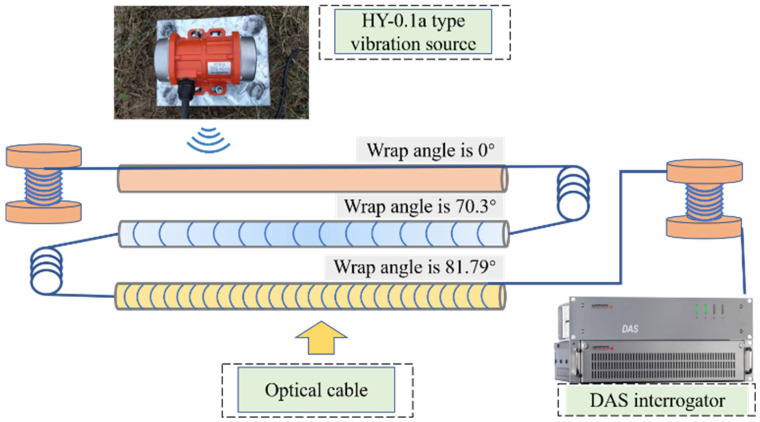
Schematic Diagram of Active Source Testing Experimental Site Layout.

**Figure 15:**
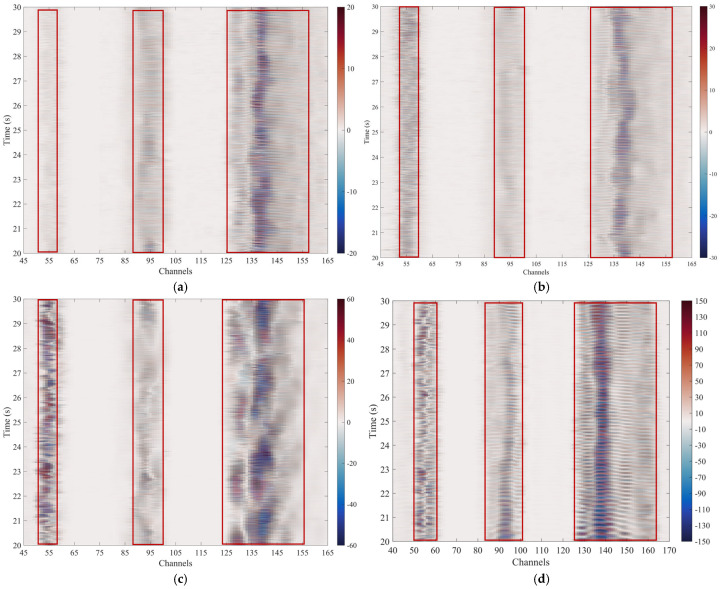
DAS measurement results of three types of optical cables under a sinusoidal vibration source. (**a**) The vibration source is 30 Hz. (**b**) The vibration source is 40 Hz. (**c**) The vibration source is 50 Hz. (**d**) The vibration source is 60 Hz.

The experimental deployment is illustrated in Figure 14 and Figure 15, where three cables with different winding angles are positioned in trenches approximately 0.5 m deep. An eccentric vibrator generates periodic vibration signals and is mounted on a base that is fully coupled to the ground surface. The periodic signals propagate through the geological strata and backfilled sandy soil to reach the test cables.

The active vibration source comprises an eccentric vibrator rotating at 3000 rpm, with frequency modulation settings of 30 Hz, 40 Hz, 50 Hz, and 60 Hz. The vibrator is secured to the ground on a base with anchor bolts, positioned 0.5 m perpendicular to the test trench. The DAS system records approximately 10 s of ambient noise before the vibrator starts, then captures roughly 10 s of signal data after the vibration stops.

### 5.2. Experimental Results Analysis

We employ an active vibration source by installing an eccentric vibrator 0.5 m perpendicular to the test trench, coupled to the ground through a base equipped with anchor bolts. The vibrator’s sinusoidal frequencies are set to 30 Hz. The DAS system records approximately 10 s of ambient noise before activating the vibration source, then records approximately 10 s of signal data before stopping.

We extracted and processed data from the signal stabilization phase. Figure 15 shows the DAS waterfall plots at frequencies of 30 Hz, 40 Hz, 50 Hz, and 60 Hz, with winding ratios of 1, 3, and 8, respectively. The horizontal axis represents the DAS channel number corresponding to the fiber length.

The results show that when the winding ratio is 1, the number of channels ranges from 53 to 57, and the actual fiber length in the cable is 5 m. Due to the spatial resolution of the DAS system and the vibration of the lead-out fiber, the measurement data exhibits broadening. Similarly, when the winding ratio is 3, the actual fiber length is 15 m, corresponding to 90 to 102 channels. In contrast, when the winding ratio is 8, the actual fiber length is 40 m, corresponding to 124 to 156 channels.

The measurement results indicate that different winding ratios produce significant differences for the same periodic signal. A higher winding ratio will result in a larger signal amplitude, which is consistent with theoretical analysis.

**Figure 16:**
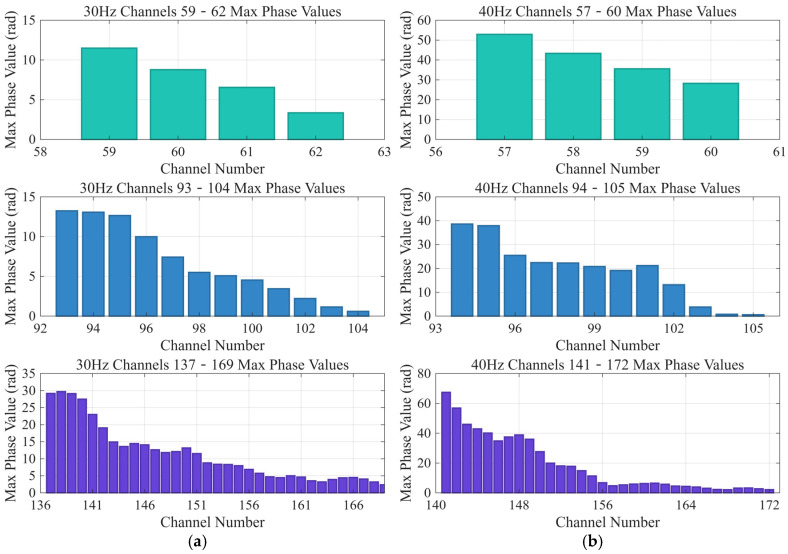

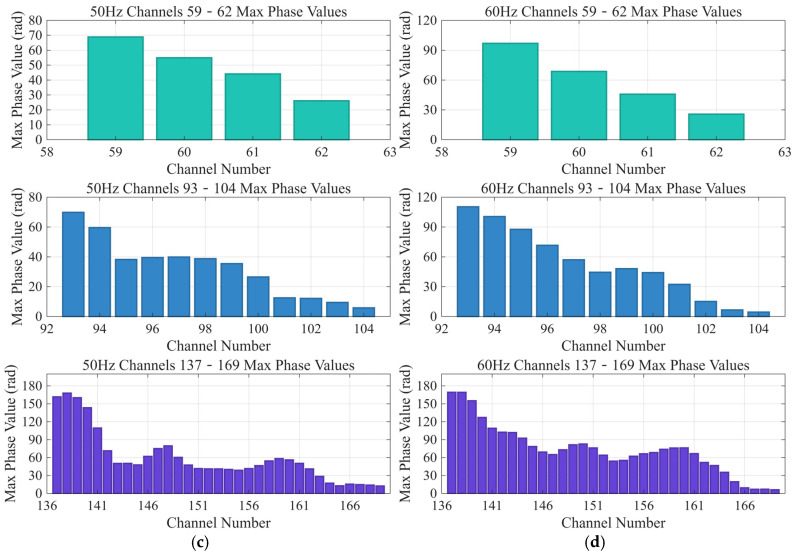
Phase attenuation of three optical cables under a vibration source. (**a**) The vibration source is 30 Hz. (**b**) The vibration source is 40 Hz. (**c**) The vibration source is 50 Hz. (**d**) The vibration source is 60 Hz.

Figure 16 shows the peak phase-attenuation trends of three optical cables with different winding angles under a vibration source, along with the phase changes for 4, 12, and 32 channels at the same length. The first panel represents a cable with a winding ratio of 1 (winding angle 0°), i.e., a straight cable. The four channels on the horizontal axis correspond to an actual sensing cable length of 5 m, based on the 1.25 m spatial sampling interval previously chosen. The vertical axis shows the peak signal phase value during the same time interval (21.5–21.6 s). Similarly, the second panel depicts a cable with a winding ratio of 3, shown with 12 channels; its wound fiber length is three times that of the straight cable. The third panel corresponds to a cable with a winding ratio of 8, also shown with 12 channels; its wound fiber length is eight times that of the straight cable.

The outdoor experiments were repeated at the exact location with vibration source frequencies of 30 Hz, 40 Hz, 50 Hz, and 60 Hz. The results show that, compared with the straight cable, wound cables with higher winding ratios (Lr) produce stronger phase responses. The spiral-winding mechanism enhances phase, markedly improving the system’s detection sensitivity.

We conducted frequency domain analysis on DAS data at 30 Hz, 40 Hz, 50 Hz, and 60 Hz, including the following main points:

(1) Multichannel Power Spectral Density (PSD) Calculation: For channels with three different winding ratios (ratios of 1, 3, and 8), we calculated their average power spectral density using the Welch method (pwelch). To ensure high-precision spectral estimation, we set a window length of 1024 points, an overlap of 512 points, and an FFT size of 2048 points.
(2) Multidimensional Visualization: We generated comparative charts, including overlaid PSD comparisons for the three ratios (0–70 Hz), subplot displays, detailed analysis of the four source frequencies, and peak power calculations.
(3) Quantitative Analysis: Peak power (dB/Hz) at the four source locations, total power across the entire frequency band, power percentage within the 20–70 Hz range, and primary frequency components were automatically extracted and presented for each ratio. This offers a quantitative basis for assessing the frequency response characteristics of different winding ratios and the energy distribution under excitation from the four sources.

This frequency domain analysis enhances the earlier time domain waterfall plots and subsequent signal-to-noise ratio (SNR) analysis by illustrating how the response strength of the target frequency signals varies across different channel setups from an energy perspective.

**Figure 17:**
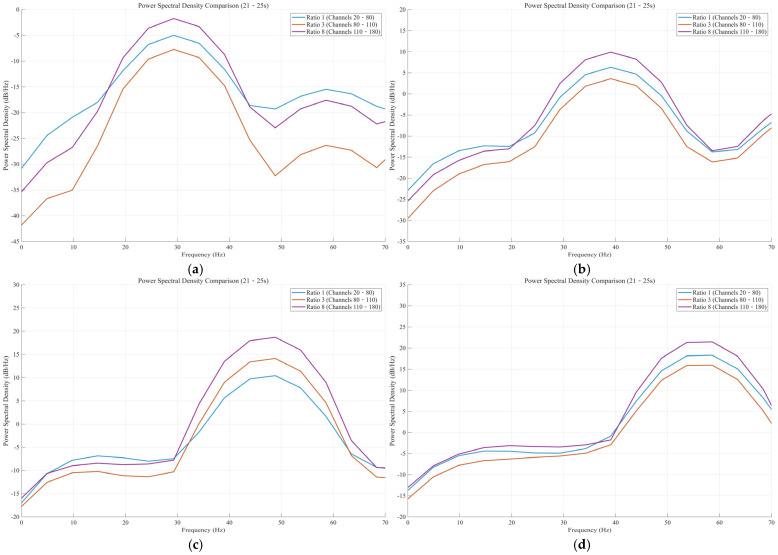
Power Spectral Density (dB/Hz) from 30 Hz to 60 Hz. (**a**) 30 Hz. (**b**) 40 Hz. (**c**) 50 Hz. (**d**) 60 Hz.

Figure 17 displays four power spectral density (PSD) plots of vibration source frequencies: 30 Hz, 40 Hz, 50 Hz, and 60 Hz. It summarizes the signal response characteristics of three configurations of optical cable, namely Ratio 1, Ratio 3, and Ratio 8, over a frequency range of 0 Hz to 70 Hz. In all of these cases, the performance of our data analysis indicates that the optical cable configuration with a ratio of 8 (Lr = 8) has the most robust signal capture capacity. Its PSD curve (purple) remains at the top in the entire spectrum, far superior to Ratio 1 (blue) and Ratio 3 (orange). The same benefit continues even at different frequencies, indicating good signal quality and the robustness of H2-T7 helical winding in Ratio 8. Notably, despite the various frequencies of the vibration source, the peak response frequency, where the PSD is highest, corresponds not precisely with the source frequency, but rather swings between 30 Hz and 55 Hz. Of these, the high peak level of PSD (∼21 dB/Hz) appears in the bandwidth characteristic of the 40 Hz source (Figure 17b), implying that during the excitation (40 Hz) the internal coupling efficiency or the response of the optical cable system is closest to a resonant frequency.

Spectral shape. All optical cables exhibited their own band-pass filter in all tests. PSD was lower in the short-term (approximately 0 Hz to 10 Hz) and high-frequency range (>65 Hz), and peaked more at mid-frequency (30 Hz to 60 Hz) in each of the experiments. The top order of optical cable performance in terms of both Ratio 8 > Ratio 1 > Ratio 3 was relatively consistent from one end to the other. The marked PSD variation between Ratio 8 and Ratio 3 is particularly high, indicating a favorable winding structure that not only increases the effective signal but also significantly reduces noise, thereby improving the signal-to-noise ratio. Additionally, Figure 17c, which represents the 50 Hz source, yields the lowest total PSD values (peak values of approximately −2 dB/Hz), indicating that the 50 Hz frequency may exhibit a low system coupling efficiency. In these less-than-ideal circumstances, Ratio 8 retained its significant advantage over the other two configurations, providing further indication of its design superiority.

**Figure 18:**
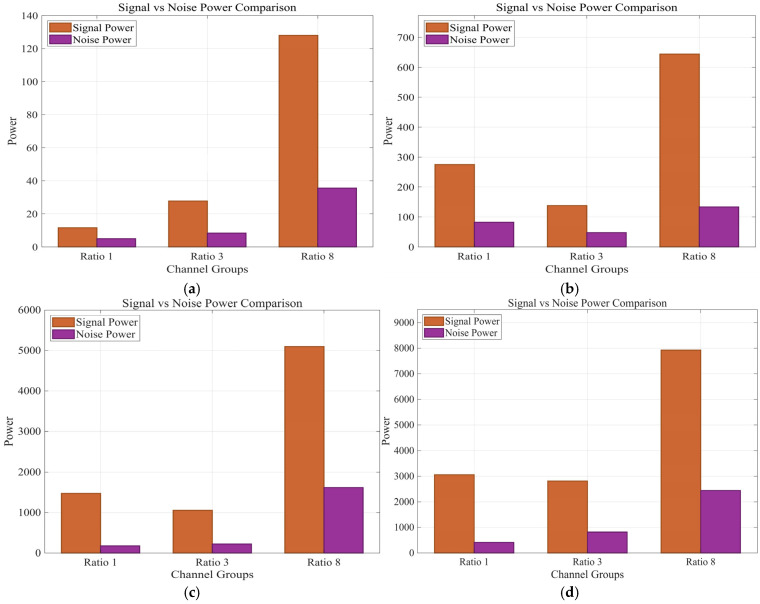
Signal vs. noise power comparison from 30 Hz to 60 Hz. (**a**) 30 Hz. (**b**) 40 Hz. (**c**) 50 Hz. (**d**) 60 Hz.

The analysis of the signal and noise power in Figure 18 reveals that the noise power curves across the four vibration frequencies intersect or are very close to each other. This suggests that variations in the excitation frequency of the vibration source have minimal impact on the noise level of the DAS system. However, when the sensing cable has the highest winding ratio, the signal power is strongest, and the noise power also rises. This suggests that the DAS signal gain originates from both the increased fiber length factor and the amplified fiber strain factor, resulting in a modest increase in noise power.

**Figure 19:**
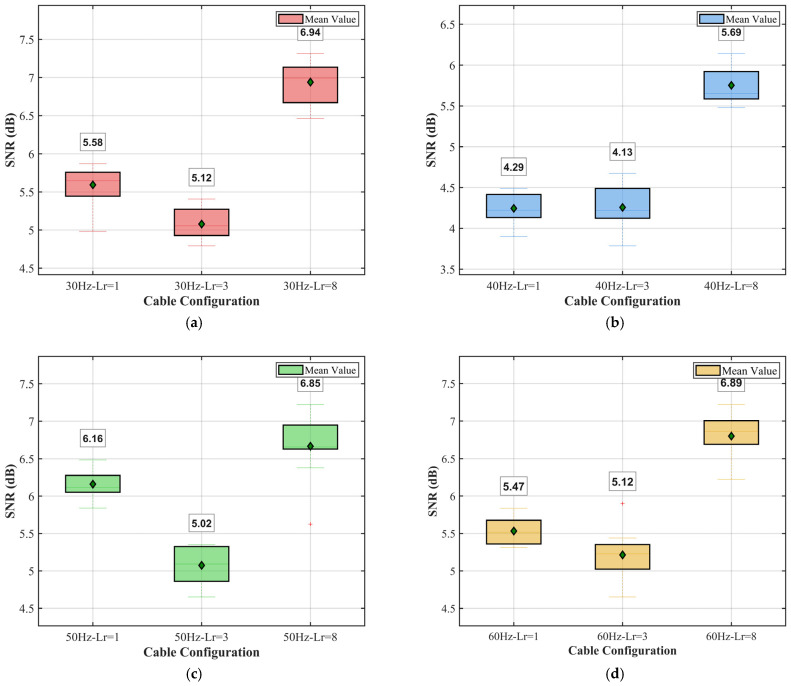
Comparison of SNRs between 30 Hz and 60 Hz. (**a**) SNR comparison at 30 Hz. (**b**) SNR comparison at 40 Hz. (**c**) SNR comparison at 50 Hz. (**d**) SNR comparison at 60 Hz.

To evaluate the repeatability and stability of the fiber-optic cable sensing system, box plot analyses were conducted for SNR measurements at four different vibration frequencies (30 Hz, 40 Hz, 50 Hz, and 60 Hz). Each frequency condition included three fiber-optic cables (Lr = 1, Lr = 3, and Lr = 8), with 10 repeated measurements for each configuration, to assess measurement consistency. A comprehensive analysis is shown in Figure 19. The signal-to-noise ratio (SNR) in decibels (dB) was calculated using the formula SNR = 10 log_10_(signal power/noise power).

At a vibration frequency of 30 Hz, a signal-to-noise ratio (SNR) is reported in Figure 19a. Cable Lr = 8 reached an SNR of 6.94 dB, while Lr = 3 reached the lowest SNR of 5.12 dB. For Lr = 1, an intermediate SNR was obtained at 5.58 dB. Using the box plot results, the SNR values of the three cables are all related, as indicated by the narrow IQR, and exhibit similar measurement performance. Moreover, the mean also aligns well with the median, as evident from the symmetric distribution and stable sensing performance. The SNR response at 40 Hz is shown in Figure 19b. In addition, the SNR values tend to be lower than those obtained in 30 Hz, where Lr = 1, Lr = 3, and Lr = 8 are 4.29 dB, 4.13 dB, and 5.69 dB, respectively. Of them all, cable Lr = 8 is shown to have the best performance at different frequencies, which indicates it is sturdy. Even at the low SNR levels at this frequency, the small size of the box plots and few outliers show that the exact measurements are extremely consistent. Figure 19c,d show the SNR distributions for all 50 Hz and 60 Hz frequencies, respectively, where the SNRs are higher for the frequencies of 40 Hz and 50 Hz, respectively. At 50 Hz, the SNRs are 6.16 dB (Lr = 1), 5.02 dB (Lr = 3), and 6.85 dB (Lr = 8). Given the frequency dependence, a sensing tool could be considered more sensitive at certain frequencies than others, depending on the resonant nature of the fiber-optic cable or the effectiveness of vibration frequency coupling when it passes through a vacuum-generating source. At 60 Hz, Lr = 8 has once again obtained the highest SNR of 6.89 dB, followed by Lr = 1 at 5.47 dB and Lr = 3 at 5.12 dB. The narrow whiskers and small IQR values for each cable and frequency confirm the high reliability and repeatability of my measurements: they are standard deviations less than 5% from the average. In general, my Lr = 8 cable consistently outperformed other configurations at all frequencies under testing; the SNR values ranged from 5.69 dB to 6.94 dB. The Lr = 3 cable always provided the lowest SNR, which can be attributed to its location or installation setup. The stable intermediate performance was obtained with the Lr = 1 cable.

The variation in SNR with frequency indicates that the optimal operating frequency for this fiber-optic sensing system is between 50 Hz and 60 Hz, where the highest SNR values were achieved. The green diamond markers near the center line in all box plots (representing the mean) further support a Gaussian distribution of the measurement noise and confirm the reliability of the experimental setup.

Analyzing from the perspective of sensing distance, a higher winding ratio results in a later drop in SNR relative to the noise baseline; therefore, Lr = 8 has the longest detectable distance. When comparing the SNR at four vibration frequencies with a fixed winding ratio, the Lr = 8 fiber-optic cable at 30 Hz has the highest SNR across all charts. Correspondingly, the Lr = 8 fiber-optic cable performs the worst at 40 Hz. A possible reason for this is that, as the frequency of the vibration source increases, the coupling curve between the fiber-optic cable and the soil decreases, leading to a simultaneous increase in both signal and noise, but a corresponding decrease in the improvement in the SNR. This again demonstrates that a high winding ratio is the key factor in improving SNR, providing the basic gain. At the same time, low frequencies represent the optimal frequency matching range for maximizing this gain.

The measured values differ from the simulated values for the vibration wave phase. The deviation is caused by variations in the attenuation coefficient at the actual outdoor test site and energy loss during wave propagation due to the coupling coefficient between the cable and the soil. However, overall, the measurement trend remains valid, and spiral-wound optical cables enhance the signal-to-noise ratio and signal strength. This phenomenon is consistent with our previous theoretical investigation and clearly points out the trade-offs involved in helical winding technology. The primary purpose of helical winding is to increase the axial strain detected by the fiber, resulting in a characteristic pattern that enhances measurement sensitivity for target vibration signals. However, this amplification process is nonselective and thus serves to boost both the target signal and environmental mechanical noise as well. While the signal is significantly improved, the noise floor has also increased, resulting in a reduced overall SNR. The main success of this experiment is that the gain from amplified effective vibration signals far surpasses the noise increase. As a result, the effective phase signal strength, once demodulated, can offset noise input and allow for accurate measurement. This strongly verifies that the design of sacrificing modest SNR in exchange for large sensitivity gain is feasible and that the experiment, as well as the associated theoretical framework, is validated.

### 5.3. Practical Challenges

Spiral fiber winding strengthens the phase response of distributed acoustic sensing (DAS) by increasing the strain caused by vibration. At the same time, however, this increase also raises some practical issues, including higher noise sensitivity (Figure 16). Both the signal gain and signal-to-noise ratio (SNR) are trade-offs that limit the scope of its applications, primarily focusing on scenarios dominated by low-amplitude events, such as shallow subsurface imaging or perimeter security monitoring. The amplified signal is used in real applications of real-time anomaly detection workflows. For instance, in pipeline integrity assessment, greater sensitivity enables the earlier detection of microseismic events, which may signify a leak or intrusion. The demodulated phase data can subsequently be processed through a threshold-based algorithm, as also reported in [24].

This allows denser virtual sensor arrays to enrich the spatial resolution of the elastic modulus of the inverted medium in geophysical exploration. The process of wavefield inversion, which minimizes the discrepancy between observed and simulated data, is at the heart of these applications, which reconstruct subsurface models. Seismic wave inversion (a common approach with high-performance DAS data such as Lr = 8 optical cables) aims at building high-precision physical parameter models of the underground medium, namely the P-wave and S-wave velocities.

This process begins with thorough data preprocessing, which includes converting raw phase data into strain rate and removing noise. Then, the distinct anisotropic response features of P-waves and S-waves in helically wound optical cables are utilized to perform a key wavefield separation step—“decoupling” the mixed recorded wavefield into pure P- and S-wave components. This step is essential for precise inversion and highlights a significant benefit of high-performance optical cables over standard ones.

The core of the inversion utilizes an advanced Full Waveform Inversion (FWI) technique, a cyclic, iterative optimization process. First, starting from an initial coarse underground velocity model (which can be obtained through traveltime tomography), theoretical seismic waveforms are calculated using numerical simulations, such as the finite-difference method. Next, this “theoretical waveform” is compared point-by-point with the high signal-to-noise “actual waveform” actually recorded by DAS, and the difference between the two is precisely calculated, known as the “residual.”

Lastly, the inversion algorithm leverages residual information to update the underground velocity model through iteration, hence ‘correcting’ it. This step aligns the waveform produced by the forward simulation more closely with the actual data. This cycle of ‘forward simulation → calculate residual → update model’ continues until the corresponding theoretical waveform is closer to that of the actual waveform with a decrease in the residual. At this stage, the model represents the final high-precision inversion result of the underground structure.

Significantly, inversion accuracy depends on these models: the normalized functions D_P_(α, Δ) and Ds(α, Δ) form the core of the DAS-specific forward modeling kernel, which accounts for helical-induced amplitude modulations and enables precise strain-to-velocity mapping. Without them, the usual straight-fiber assumptions would cause biased inversions, underestimating responses in all directions. Overall, although challenges remain in noisy environments that need adaptive regularization, this method improves DAS’s potential for quantitative media analysis in engineering applications.

## 6. Conclusions

In this paper, we systematically approach the nonlinear features of DAS systems for seismic wavefield detection methods and propose a solid methodology for parameter optimization and structural design. We first clarify the relationship between gauge length and pulse width to specify their spatial filtering effect on vibrational waves, and then establish their importance for obtaining accurate and useful results in velocity inversion. Second, we formulate trigonometric response models for P- and S-waves at various incidence angles (i.e., these models are used to evaluate fiber sensitivity). Finally, we develop and validate a sensitivity enhancement model of helically wound cables, finding that larger winding angles increase the integration range, make measurements more sensitive, and depend on factors such as source distance, site conditions, and wave frequency. In addition, this research not only establishes a link between optical phase demodulation and the inversion of geophysical parameters but also addresses the practical tradeoff of increased noise in high-sensitivity systems. By enabling denser virtual arrays and improved SNR in low-amplitude environments, this advances DAS applications in geophysics, pipeline integrity, and structural health monitoring. Future work should include advanced simulations to reduce fatigue in helical designs and extend models to anisotropic media, promoting wider adoption in cutting-edge sensing technologies.

## Figures and Tables

**Table 1:** SNR analysis results.

Winding Angle	Winding Ratio	SNR(dB)	Maximum Phase Amplitude (rad)
0°	1	2.87	1.071
70.3°	3	15.04	3.238
78.46°	5	20.30	9.581
81.79°	7	23.23	12.849

## Data Availability

The data that support the findings of this study are available from the corresponding author upon reasonable request.

## Abbreviations

The following abbreviations are used in this manuscript:

α	Local angle in sensitivity decomposition.
β	Helical winding angle
δ	Incidence angle of the wave relative to the fiber axis
Δϕ	Change in optical phase due to strain
ϕ-OTDR	Phase-sensitive optical time domain reflectometry
$\varepsilon_{z}$	Axial strain along the fiber
$\varepsilon_{z}^{DAS}$	Effective strain after spatial averaging
$\varepsilon_{zz}$	The actual axial strain component along the fiber direction
θ	Helical winding angle of the fiber
$\;\lambda_{c},\lambda_{g}$	First Lamé parameters for cable and geological formation
ω	Angular frequency of the wave
$A_{0}$	Amplitude of the incident wave
DAS	Distributed acoustic sensing
$D_{P}$	Sensitivity components
$D_{S}$	Effective refractive index of the fiber mode
GL	Gauge the length of the DAS system.
k	Wave number of light
$k_{z}$	Axial wavenumber component
$L_{c}$	Actual length of helically wound fiber over one pitch
$L_{f}$	Axial pitch length of the cable core
n	Refractive index of the optical fiber
$N_{c},{\;N}_{g}$	Shear moduli for cable and formation
OTDR	Optical time domain reflectometry
$P_{11}$$,P_{12}$	Photoelastic coefficients of the fiber
P-wave	Primary wave
S	Total sensitivity of the helically wound fiber
S-wave	Secondary wave
SH wave	Horizontally polarized Shear wave
SV wave	Vertically polarized Shear wave
SNR	Signal-to-noise ratio
$z^{\prime }$	Integration variable along the fiber axis
